# Bioinformatic flowchart and database to investigate the origins and diversity of Clan AA peptidases

**DOI:** 10.1186/1745-6150-4-3

**Published:** 2009-01-27

**Authors:** Carlos Llorens, Ricardo Futami, Gabriel Renaud, Andrés Moya

**Affiliations:** 1 Institut Cavanilles de Biodiversitat i Biología Evolutiva, Universitat de València, Paterna, Valencia, Spain; 2 Biotechvana, Parc Cientific, Universitat de Valencia, Paterna, Valencia, Spain; 3 CIBER de Epidemiología y Salud Pública (CIBERESP), Barcelona, Spain

## Abstract

**Background:**

Clan AA of aspartic peptidases relates the family of pepsin monomers evolutionarily with all dimeric peptidases encoded by eukaryotic LTR retroelements. Recent findings describing various pools of single-domain nonviral host peptidases, in prokaryotes and eukaryotes, indicate that the diversity of clan AA is larger than previously thought. The ensuing approach to investigate this enzyme group is by studying its phylogeny. However, clan AA is a difficult case to study due to the low similarity and different rates of evolution. This work is an ongoing attempt to investigate the different clan AA families to understand the cause of their diversity.

**Results:**

In this paper, we describe in-progress database and bioinformatic flowchart designed to characterize the clan AA protein domain based on all possible protein families through ancestral reconstructions, sequence logos, and hidden markov models (HMMs). The flowchart includes the characterization of a major consensus sequence based on 6 amino acid patterns with correspondence with Andreeva's model, the structural template describing the clan AA peptidase fold. The set of tools is work in progress we have organized in a database within the GyDB project, referred to as Clan AA Reference Database .

**Conclusion:**

The pre-existing classification combined with the evolutionary history of LTR retroelements permits a consistent taxonomical collection of sequence logos and HMMs. This set is useful for gene annotation but also a reference to evaluate the diversity of, and the relationships among, the different families. Comparisons among HMMs suggest a common ancestor for all dimeric clan AA peptidases that is halfway between single-domain nonviral peptidases and those coded by *Ty3/Gypsy *LTR retroelements. Sequence logos reveal how all clan AA families follow similar protein domain architecture related to the peptidase fold. In particular, each family nucleates a particular consensus motif in the sequence position related to the flap. The different motifs constitute a network where an alanine-asparagine-like variable motif predominates, instead of the canonical flap of the HIV-1 peptidase and closer relatives.

**Reviewers:**

This article was reviewed by Daniel H. Haft, Vladimir Kapitonov (nominated by Jerry Jurka), and Ben M. Dunn (nominated by Claus Wilke).

## Background

Clan AA of the aspartic peptidases (CAPs) [1] is a group of proteolytic enzymes that use an aspartate dyad and a molecule of water to hydrolyze a peptide bond [2]. There are 2 major forms of this enzyme; the single domain aspartic peptidase (LTRCAP) encoded by eukaryotic LTR retroelements, which dimerizes in its active form and the 2-domain pepsin monomer, involved in the metabolism and proteolysis of food by eukaryotes. Because of the similar pseudo-symmetry it is normally assumed that pepsins evolved from the duplication [3] of an ancestral dimeric form similar to retroviral proteases [4] (i.e. LTRCAPs). This scenario is supported by recent issues [5-9] describing 2 related families of single-domain nonviral peptidases in prokaryotes (pSNCAPs) and 3 others (eSNCAPs) in eukaryotes. However, the split between the 2 structural forms is probably older than previously thought as sequencing projects have recently revealed the presence of pepsin representatives in several prokaryotic genomes (annotations available at MEROPS [10]). Little is known about pSNCAPs (COG3577 and COG5550), which are open reading frames (ORFs) of ~120 residues widely distributed in α-proteobacteria [5] and also present in archaea such as *A. fulgidus *(Genbank accession AAB90625). In contrast, the 3 eSNCAP families namely as SASPases, DNA-damage inducible(DDI) peptidases, and neur onal interacting factor × 1 (NIX-1), have been studied more extensively. DDI peptidases are enzymes of ~350 residues in length, widely distributed in plants, fungi and animals [5] and exhibiting a central CAP domain flanked by ubiquitin and ubiquitin-associated domains [5,7]. The recent characterization of the 3D structure of this enzyme confirms that it is a dimer with a similar fold to LTRCAPs [6]. NIX-1 is a nuclear receptor-associated protein of ~250 residues [11] that preserves a CAP domain closely related to that of DDI [5,7]. SASPases constitute a set of host enzymes of ~280 residues, displaying a single CAP domain toward the C-terminus. SASPases are specifically expressed in at least human and mouse epidermis [8,9], and have been implicated in several dermal side effects induced by protease (i.e. peptidase) inhibitors in anti-AIDS therapy [9].

The derived approach to investigate the relationships between the new and the old clan AA members is by studying their diversity and phylogeny. However, the evolutionary history of clan AA is extremely difficult to assess. Retroviruses and LTR retrotransposons are not under the same pressure of error correction that affects host genes, the mutation rates of LTR retroelements probably exceed those of their nonviral counterparts. The large diversity of eukaryotic LTR retroelements and encoded CAP products creates a daunting task when trying to establish relationships and make classification. Upon that, it is currently known that the most representative LTR retroelements inhabiting the eukaryotic genomes can be divided in 5 groups – *Ty3/Gypsy, Ty1/Copia, Bel/Pao, Retroviridae *and *Caulimoviridae *(for more details, see [12]). However, the different CAPs encoded by these groups have proved to be hard to keep track of in eukaryotic genomes, as most of these sequences remain unclassified or have conflicting phylogenetic signal (searches for potential homologues using LTRCAP queries produce too many hits, most of which are pseudogenes or deactivated retrotransposons).

An important principle of peptidase classification has been established by MEROPS [1] (a general database on enzymology), according to which, pepsins and LTRCAPs can be divided in five families: A1, A2, A3, A9 and A11 [1]. In this classification, pepsins represent the family A1, which splits in 2 subfamilies – A1A and A1B. The different LTRCAPs encoded by vertebrate retroviruses (*Retroviridae*) are classified as the family A2 (retropepsins) except those encoded by spumaretroviruses, which were assigned to the family A9 (spumaretropepsins). The LTRCAPs encoded by caulimoviruses and *Ty1/Copia *LTR retroelements have been assigned to the families A3 and A11, respectively. MEROPS also classifies a few examples of Ty3/Gypsy LTRCAPs in several sub-families within the family A2 because of their similarity to retropepsins. However, not all Ty3/Gypsy LTRCAPs are similar to retropepsins just as not all the Retroviridae LTRCAPs are retropepsins. In fact, the diversity in Ty3/Gypsy LTRCAPs greatly exceeds their current classification. LTRCAPs encoded by *Bel/Pao *LTR retroelements [12-14] and the different SNCAP families of prokaryotes and eukaryotes have no current family classification. Beyond MEROPS, there are no studies addressing an exhaustive sequence evaluation of clan AA in the post-genomic era. Having recognized that, the large diversity of eukaryotic LTR retroelements revealed by sequencing projects clearly justifies the development and continuous update of a curated database focusing on the investigation and classification of the different LTRCAP families and their related host genes.

In this paper, we introduce the Clan AA Reference Database (CAARD), a phylogenetic database hosted at Gypsy Database (GyDB) Project [15] and developed with the aim to classify the different clan AA families according to different estimations of their taxonomy and phylogeny. The characterization addressed here is compatible to, but suggests an improvement over the pre-existing classification, as we consider the evolutionary history of LTR retroelements and the recently reported SNCAPs. Thus, we will be receptive to all MEROPS suggestions to assign family letters to the different families described in this and further CAARD versions. In fact, the approach is in progress because the actual diversity of clan AA cannot be realistically determined in a single study. In this first version, we perform an evaluation of all clan AA families, but pay particular attention to Ty3/Gypsy and Retroviridae LTRCAPs according to their differentiation into clades and genera. The main objective of this first CAARD version is to investigate the major consensus and phylogeny to typify the different protein families by sequence logos and HMMs. With this aim, we designed a flowchart of bioinformatic analyses to confirm and standardize the phylogenetic signal of each family using maximum likelihood reconstruction (AMLR) before to its characterization by sequence logos and HMMs. The flowchart is detailed online as an additional section of CAARD. Finally, we use the set of sequences logos and HMMs to perform here a comparative approach to explore certain aspects of clan AA related to the evolutionary history and diversity of this enzyme group.

## Results and discussion

### Clan AA families and bioinformatic flowchart

This work is an attempt to characterize the clan AA protein domain in all its possible phylogenetic signals. With this goal, we combined the pre-existing classification [1] with the International Committee on Taxonomy of Viruses (ICTV) [16] and prior studies [5-9,12,15,17], to assign taxonomy levels to 323 non-redundant canonical CAPs (Table 1). Here, we recognize the 2 pepsin subfamilies A1 and A2 at MEROPS [1] but for analytical purposes, we divided these 2 into 4 families by considering each pepsin domain as a separate family. Our classification is work in progress. In this first version, we pay particular attention to the classification of Ty3/Gypsy and Retroviridae LTRCAPs according to their differentiation into clades and genera of LTR retroelements. This classification derives from prior studies and an exhaustive investigation of the 2 groups based on not only the CAP domain but also on other protein domains (see [15,18]). In phylogenetic terms, the current classification is not a particular bias against other LTRCAPs such as those encoded by *Ty1/Copia *and *Bel/Pao *LTR retroelements because they have phenotypic features distinguishing them from other CAPs. Despite this, *Ty1/Copia *and *Bel/Pao *LTR retroelements are also rich in lineages and variability. In further versions, we are committed to perform similar investigation based on these and other LTR retroelement groups to revise the existing clan AA families in light of new data.

**Table 1:** Clan AA aspartic peptidases: taxonomy, families and sequences

taxonomy	families	MEROPS	seqs	citations
Retroviridae LTRCAPs	Lentiviridae	A2A	11	[16]
	Alpharetroviridae	A2A	3	
	Betaretroviridae	A2A	8	
	Gammaretroviridae	A2A	13	
	Deltaretroviridae	A2A	4	
	Epsilonretroviridae	A2A	1	
	Spumaretroviridae	A9	6	
	MuERV-L	No-letter	1	

Ty3/Gypsy LTRCAPs	412/mdg1	No letter	2	[[15], and references therein]
	Athila	No letter	9	
	Cer1	No letter	1	
	Cer2-3	No letter	2	
	Chrofung*	No letter	14	
	CsRN1	No letter	2	
	CRM	No letter	3	
	Del	No letter	7	
	Errantiviridae	A2C and 2G	14	
	Galadriel	No letter	3	
	Mag	No letter	7	
	Micropia/mdg3	No letter	3	
	Osvaldo	A2D	4	
	Reina	No letter	4	
	Tat	No letter	10	
	TF1-2	A2E	2	
	Ty3	A2B	3	

Other LTRCAPs	Bel	No letter	16	[1,12,17]
	Bs-1	No letter	1	
	Caulimoviridae	A3	18	
	Ty1/Copia	A11	26	

pSNCAPs	COG5550	No letter	10	[5]
	COG3577	No letter	20	

eSNCAPs	DDI	No letter	20	[5-9]
	NIX-1	No letter	5	
	SAPases	No letter	6	

Pepsins	domain-1	A1A**	27	[1]
	domain-2	A1A**	27	
	domain-1	A1B**	5	
	domain-2	A1B**	5	

*The descriptor "Chrofung" collects the CAPs encoded by *Ty3/Gypsy *chromoviruses of fungi and vertebrate organisms.**In this study, each pepsin subfamily at MEROPS is represented by 2 families, one per each protein domains (1 and 2 or N- and C-terminal). For information about the Genbank accessions, see the Additional file 1 accompanying online this paper.

**Figure 1:**
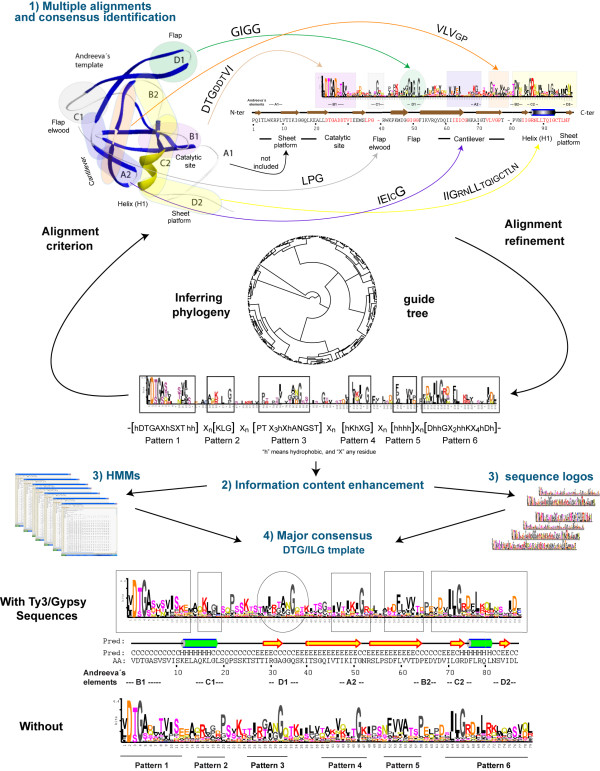
**Flowchart**. This work is an attempt at investigating the diversity of the clan AA protein domain based on all its possible families. With this goal, we designed a flowchart of bioinformatic analyses to regularize and characterize the different phylogenetic signals. The flowchart consists of 4 steps: 1) Multiple alignments and consensus identification; 2) Information content enhancement; 3) HMMs and sequence logos; 4) Major consensus.

The most important difficulty of clan AA is that the families considered here share less than 20% of identity and follow different phylogenetic signals. This makes it very difficult to establish conclusions about similarity, major consensus and evolutionary relationships of the different families summarized in Table 1. With this aim, we designed a bioinformatic flowchart using manual means and software analyses to standardize and characterize the different phylogenetic signals. The flowchart scheme is presented in Figure 1 and we provide extensive description of the flowchart in an additional section of CAARD available online at [19]. The flowchart consists of 4 steps – multiple alignments and consensus identification, information content enhancement, HMMs and sequences logos, and major consensus.

The first step involves the creation of 34 multiple alignments, one for each family including 2 or more CAPs to characterize the protein domain architecture of each family (we dismissed the LTRCAPs representing one-sequence families because they cannot be established in family alignments). Additionally, we performed a non-redundant single multiple alignment with all CAPs to investigate the phylogeny and major consensus. Here, we used prior structure-based alignments [4,20] and the Andreeva's model [21] as the criterion to manually align the different families. The alignment was refined using the guide tree illustrated in Figure 1 (an extended version of this tree is available online, as the Additional file 1). That is, prior structure-based comparisons between retropepsins and pepsins revealed a common hydrophobic core of 90–190 residues [4,20]. This core can only be used to describe the similarity between 2 or 3 families, due to the low amino acid identity among families. In an attempt to establish a universal reference, Andreeva presented in 1991 a structural template to describe the peptidase fold of pepsins [21] and showed that retroviral LTRCAPs also fit into this template (for a more detailed overview of pepsins, see [22]). The clan AA peptidase fold displays 6 major regions, namely the catalytic site, the sheet platform, the helix (H1), the cantilever, the flap elwood, and the flap (all emphasized in colors in Figure 1). These regions are the result of the physiochemical interactions of minor structural elements which Andreeva described as follows: a N-terminal loop A1, a loop B1 containing the catalytic motif, an α-helix C1 not preserved by several LTRCAPs, a β-hairpin loop D1, a hairpin loop A2, a wide loop B2 containing the ILG motif B2, an α-helix C2 and a loop D2 substituted in several LTRCAPs by a strand or a helical turn. Subsequent issues confirmed and expanded this model to all enzymes belonging to clan AA (see [23]). Because of the low similarity, there are no sequences related to Andreeva's model in previous studies, except retropepsins and pepsins. However, while performing the non-redundant alignment, we identified 6 amino acid patterns with structural correspondence with the Andreeva's model [21]. Thorough the rest of this paper, we will refer to the 6 amino acid patterns as the DTG/ILG template because the 2 canonical DT/SG and ILG amino acid motifs [4,24] are prominent in this template. Figure 1 shows the correspondence between the DTG/ILG template and the Andreeva's model by using lentiviral CAPs as a canonical retropepsin-like example (for more details on this topic see [23,25]). The DTG/ILG template provides not only the needed criterion to align exhaustively the different clan AA families but also a major consensus based on the Andreeva's model. We tried to characterize this template as an HMM and a sequence logo using the non-redundant alignment as an input to different tools. However, all tools failed to reconstruct informative material because of the variability and multiple gaps introduced in the alignment. At least, we were able to approximate a preliminary sequence logo shown in the center of Figure 1, which is not significant under conventional logos methodology but is sufficiently informative to visualize the major consensus sequence (in the words of George P. Box quoted by Schneider [26] – "all models are wrong, but some are useful").

The second step considers the information content enhancement of each family alignment by AMLR. We included this analysis in the flowchart because many families have conflicting signal and because the degree of sequence preservation varies depending on the family. In other words, the object of the AMLR analysis was to increase prominent sequence patterns specifically preserved by each set of monophyletic CAPs.

In the third step, we chose the AMLR alignments obtained with the Jrof method to create a collection of sequence logos and HMMs modeled as HMM profiles and as majority-rule consensus (MRC) sequences.

In the fourth step, we reconstructed the DTG/ILG template. Noting that the previously resolved sequence logo of this template is only an approximation, we completed a more precise characterization using a master alignment. Here, we anchored the different MRCs derived from HMMs and 4 LTRCAPs representing one-sequence families to perform an additional AMLR analysis in 2 different ways (with and without Ty3/Gypsy sequences). Subsequently, we used the 2 Jrof alignments reported by the 2 AMLR analyses to characterize the DTG/ILG template as an HMM and as a sequence logo. Figure 1 also shows the 2 alternative sequence logos constructed using Shannon's algorithm [27] and a correction factor for < 100 sequences. Both logos exhibit similar information content shape, which suggests that the template is not biased toward Ty3/Gypsy or any sequence in particular, but that its information content shape derives from sequence patterns imprinted by the peptidase fold. The 2D structure below of the DTG/ILG temple sequence logo describes the canonical "helix-strand-helix" template (i.e. the Andreeva's model) usually resolved and/or approximated based on predicted and/or empirical data. The 2 alternative DTG/ILG template forms thus suggest that Andreeva's model is reproducible by sequence not only using the same material employed here, but also with other subsets of non-redundant sequences.

### The database

**Figure 2:**
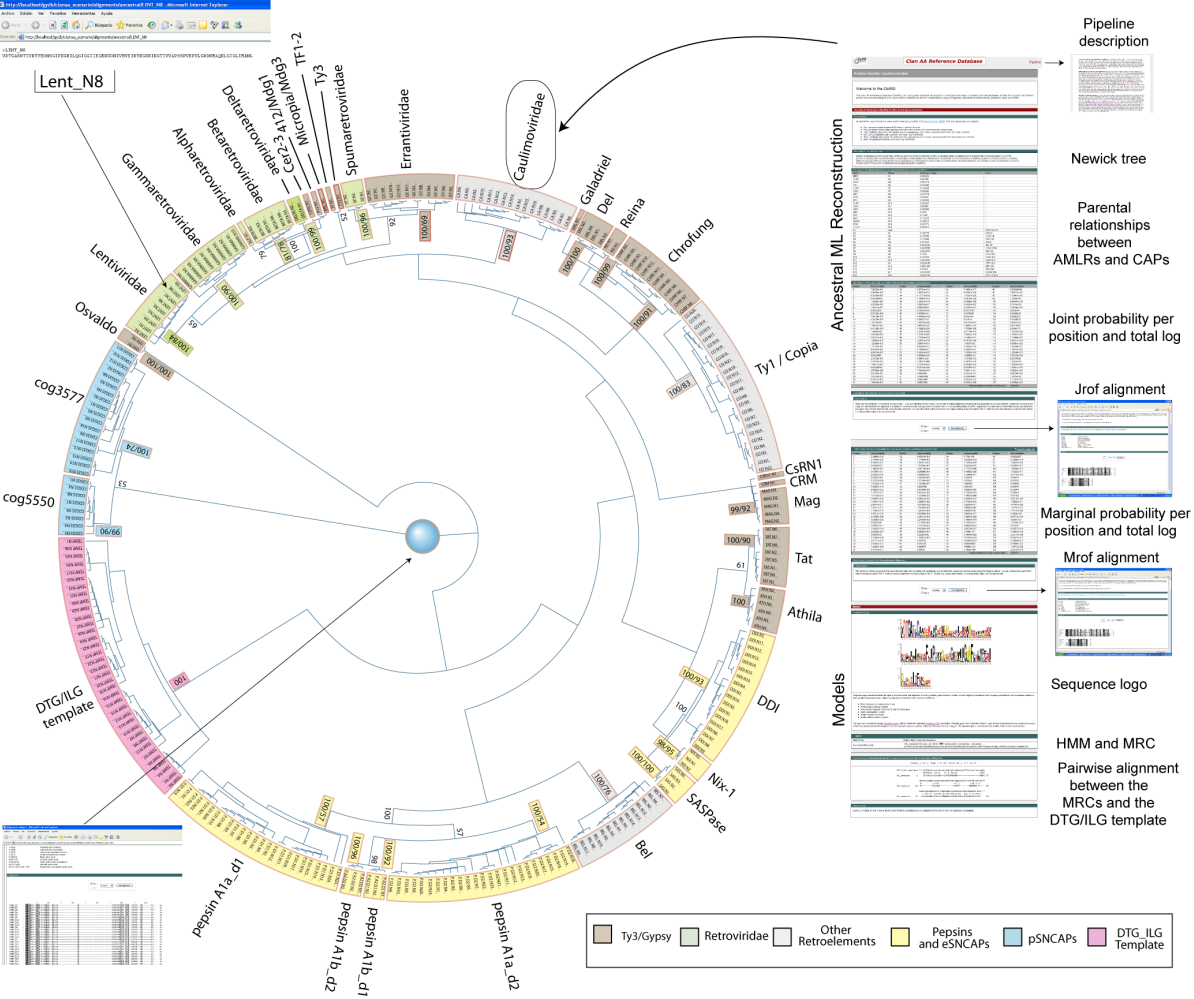
**Database screenshot**. We stored the set of tools created in this study in an ongoing database referred to as CAARD. The database can be navigated via a phylogenetic tree, inferred based on all processed Jrof AMLR sequences. The tree is dynamic and allows the user to retrieve information from the database in different ways. Colored features link to the different datasheets stored, we have divided in 2 main sections – "Ancestral ML reconstruction" and "Models". Each section is divided in subsections as indicated in the Figure.

The most valuable feature here is the set of alignments, AMLRs, sequence logos and HMMs. This set is in progress because the actual diversity of clan AA cannot be determined in a single study. With this motivation, we organized the entire collection in CAARD, except the original alignments, which are freely accessible in various formats within the GyDB collection [28] deposited at Biotechvana Bioinformatics [29] (for accessing the alignment URLs, see the additional file 2 accompanying online this paper). As shown in Figure 2, CAARD is based on a number datasheets, one for each family plus another one focusing on the DTG/ILG template. The datasheets are structured into 2 main sections – "Ancestral ML reconstruction" and "Models". The first includes 6 sub-sections, one for each output generated by the AMLR analysis. This includes Newick tree (the most commonly accepted format to represent phylogenetic trees using parentheses and commas), statistics data and values related to the AMLR, as well as the 2 Jrof and Mrof alignments available in various formats. The user can download the Jrof and Mrof AMLR alignments in 2 ways, processed or unprocessed (as the output were originally reported by the AMLR analysis, see "Methods"). The second section contains the sequence logo and the HMM profile and its derived MRC sequence. This section also provides a pairwise alignment between the MRC and the DTG/ILG template to facilitate a comparison between the major consensus and each particular family consensus. By default, the datasheet focusing on the DTG/ILG template presents the material derived from characterization using Ty3/Gypsy sequences. This site links to another datasheet, which supplies the alternative characterization performed without Ty3/Gypsy information. Upon the database, we are working to implement the next version using wiki software to let other authors to contribute material such as structures, more HMMs, and/or consensus sequences or position-specific scoring matrices (PSSMs) iterated for instance, from specialized databases such as MEROPS [1] and REPBASE [30] or from genome projects.

The database is accessible through a dynamic tree the user can move, resize and rotate in different ways. By clicking the name of each cluster in this tree, the user can locate the datasheet corresponding to the selected family. The tree has been inferred based on the single non-redundant alignment of all Jrof AMLR sequences reconstructed in this study. The sequences can be retrieved in 3 independent ways: 1) within each data sheet as a Jrof alignment, 2) as a non-redundant alignment by clicking on the blue circle at the center, 3) in separate files clicking the leaves of the tree. Here, the different AMLR sequences have tags with information about the parental relationships of the sequence represented by each leaf. Note the example provided at the top of Figure 2, where the abbreviation "LENT" indicates "lentiviral family" and the tag "N_8" denotes the reconstructed node represented by the selected sequence. To infer the tree we used the parsimony method of phylogenetic reconstruction [31,32], but other methods such as neighbor joining (NJ) [33] equally support the families. Values accompanying the clusters are bootstrap estimations supporting the clusters (families) that occurred > 55% of the time in the parsimony and NJ analyses. In general, the tree confirms the different families summarized in Table 1 and shed light on the relationships of Ty3/Gypsy LTRCAPs with other families. The tree shows how, depending on the family, Ty3/Gypsy LTRCAPs can be similar to other LTRCAP families or to SNCAPs. At this point, the current tree has also reasonable limitations that we are committed to resolve in further versions. In particular, the tree shows certain ambiguities due to the conflicting signals between certain families, and we also think that a more extensive differentiation of Ty1/Copia and Bel/Pao LTRCAPs into families will probably modify the internal branches of this tree in further versions.

### Database topic: Investigating the diversity and relationships among the different clan AA families through sequence logos and HMMs

The set of tools deposited in CAARD provides various methods and resources to evaluate the diversity of, and the relationships among, the CAPs of LTR retroelements and their homologous host genes of eukaryotes and prokaryotes. The most obvious use of this set is for gene annotation. Along these lines, we are currently testing the different HMMs and MRCs with the *Pea aphid *genome, where they have proved excellent for detecting and annotating new *caps *ranging from pepsins and *ddi *host genes to different LTR retroelement lineages (manuscript and database update in preparation). In this section, we show other CAARD applications performing a comparison of sequence logos and HMMs to investigate certain aspects of clan AA related to its evolutionary history and diversity. The qualitative comparison of all sequence logos deposited in CAARD reveals that any clan AA family approximates similar information content shape we divided in 6 amino acid patterns with structural correspondence with Andreeva's model. This suggests that, with obvious conformational variations, all CAPs investigated follow similar peptidase fold architecture. One of the most interesting aspects of this fact is the consistency between the DTG/ILG template's pattern 3 and the flap the structural β-hairpin loop that covers the active site carrying substrate-binding functions (see [23]). In a prior study [18], we investigated the relationships of *Ty3/Gypsy *and *Retroviridae *LTR retroelements based on the differentiation of vertebrate retroviruses into 3 previously reported classes [34-38]. What we found is that 3 protein products (one of them being the CAP) exhibit phenotypic differences we used to describe an evolutionary network between *Ty3/Gypsy *and *Retroviridae *LTR retroelements. Regarding the CAP, the network involves 3 isoforms of the enzyme showing 3 flap variations, namely as GIGG, GANG and TIHG [18]. We chose this nomenclature because of the tendency of pattern 3 to nucleate particular consensus motifs with similar physiochemical properties, depending on the *Retroviridae *class and *Ty3/Gypsy *clade or genus. The GIGG variation defines the well-known glycine-rich flap of the LTRCAP encoded by HIV-1 and other class II relatives (see [23]). We observed that several Ty3/Gypsy clades code for LTRCAPs preserving this variation. The GANG motif is an idealized consensus used to describe a variation usually characterized by the predominance of an alanine and an aspartate/asparagine/threonine in the equivalent flap trait of almost all LTRCAPs encoded by the *Retroviridae *class I (epsilon- and gammaretroviruses). Along these lines, we found that other *Ty3/Gypsy *clades codify for LTRCAPs bearing this variant. Additionally, we found that the TIHG variant defines the flap [39] of the LTRCAPs coded by class III spumaretroviruses, and that many but not all *Ty3/Gypsy *elements belonging to the genus *Errantiviridae *encode for LTRCAPs preserving this variation.

**Figure 3:**
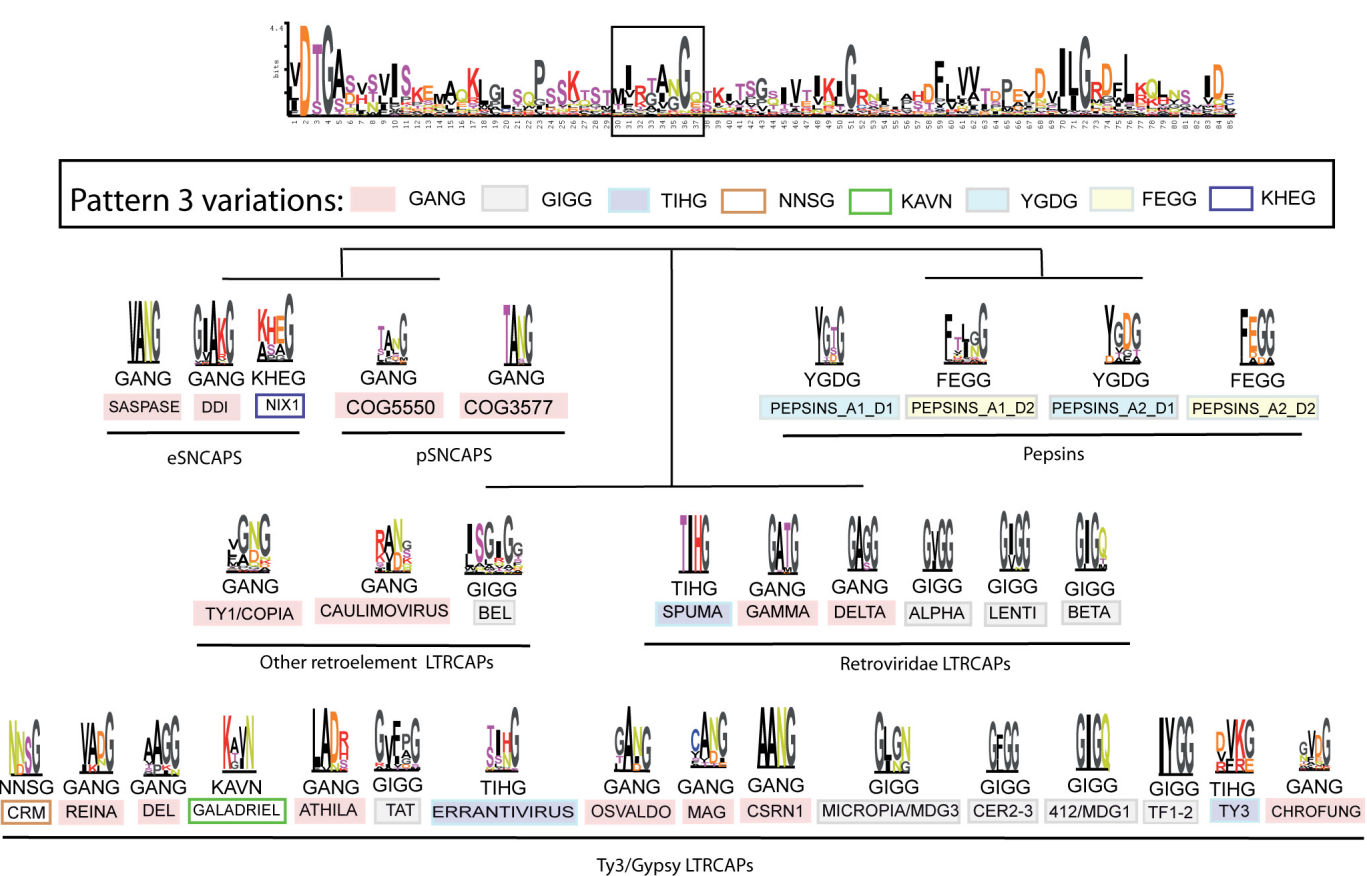
**Pattern 3 variations**. The DTG/ILG template's pattern 3 defines the sequence position equivalent to the β-hairpin loop called flap. A comparison among all constructed sequence logos reveals how, depending on the family, this position nucleates particular consensus motifs related but not identical to those of other families. We categorized this network into 8 variants according to the physical-chemical properties of residues and only show the entire sequence logo of the DTG/ILG template (the remaining logos are available within the database under "Results"). In this network, an alanine-asparagine variable motif predominates among LTR- and SNCAPs, instead of the canonical glycine-rich motif of HIV-1 peptidase and closer relatives.

Regarding clan AA, the logos comparison reveals that not only Ty3/Gypsy and Retroviridae LTRCAPs but also all other clan AA families nucleate intrinsic consensus motifs in pattern 3. As shown in Figure 3, the different motifs are similar but not identical each other. We show only 4–6 residues of the most prominent of each pattern 3 motif delineated by the information content shape of each sequence logo. As this scheme extends the Ty3/Gypsy/Retroviridae network to all other CAPs, we categorized the different motifs into 8 idealized variations following the GANG-GIGG nomenclature introduced in [18]. The flap-pattern 3 equivalence is supported by empirically characterized data. The 3D structures available of the CAPs encoded by SFV [39], HIV-1 [40], HTLV-1 [41,42] retroviruses and DDI peptidases [6] are indistinctly and clearly delineated by 3 different TIHG, GIGG, and GANG motifs. Although the domain-2 of pepsins does not have a true flap, the pepsin structures empirically characterized also support the equivalence between pattern 3 and the flap of the pepsin domain-1 (see the PDB-files accessions available at MEROPS). Finally, the motif's similarity between the empirically characterized flap and the highly preserved pattern 3 found in almost every, but not all, sequence logos suggests that the likeliest function for pattern 3 is flap. Upon that, the CAPs belonging to families exhibiting a highly preserved pattern 3 usually show the same motif. The best alignment for this motif when aligning (family-to-family and by both automated and manual means) any of these families with retropepsins is consistent with the flap motif. This suggests no bias in the alignment of these families. However, Figure 3 also evidences how the preservation of pattern 3 varies depending on the family and that many families characterized show poorly preserved pattern 3 motifs. This could be due to the following: 1) several families may be carriers of 2 or more pattern 3 variants; 2) the flap may be able to evolve from one state to another within and between families; 3) many CAPs might have lost the flap as the pepsin-domain-2 probably did. These possibilities are not mutually exclusive and by this reason we do not dismiss putative bias in the sequence logos of poorly preserved families toward the most predominant pattern 3 motif (as a consequence of the enhancement of patterns performed by the AMLR analysis). This fact probably has a negligible effect to the overall biological conclusions but suggests that families showing poorly preserved pattern 3 motifs may enclose more protein isoforms, which should be characterized by separate HMMs and logos to clarify all possible pattern 3 variants.

One interesting aspects of the network is that it suggests that pattern 3 is an evolutionary marker helpful in tracing several aspects of both clan AA and LTR retroelement evolution. In this particular, the GANG variant is prominent in the DTG/ILG template sequence logo. This is because this variation is more frequent in the network than any other variation (such as the well-known GIGG motif of the HIV-1 LTRCAP and relatives). Speculating, this suggests that the GANG variation is probably the ancestral state from which other variants evolved, in at least dimeric CAPs. Upon that, the qualitative similarity between various GANG motifs derived from the LTR- and SNCAP families is consistent with the CAARD phylogeny and suggests a putative relationship between LTR- and SNCAPs. Evaluation of the sequence logos dismisses Ty1/Copia and Bel/Pao LTRCAPs from this relationship because these 2 families exhibit phenotypic features distinguishing them from other CAPs. Among other features, Ty1/Copia LTRCAPs usually preserve an ILS motif substituting the ILG motif displayed at the C-terminus of almost all other CAPs (material available in CAARD). In similar terms, Bel/Pao LTRCAPs present an extremely large and variable core that is rich in serine-threonine residues (material available in CAARD). In contrast, caulimoviruses, *Ty3/Gypsy *and *Retroviridae *LTR retroelements code for a variety of CAPs, which are similar to SNCAPs based on sequence and protein domain architecture. To investigate which of these 3 LTRCAP families is more similar to SNCAPs, we performed additional analyses comparing all HMMs to each other. We do not discuss all cases, only the most significant instances in performing the analysis – Ty3/Gypsy LTRCAPs and SNCAPs. As shown in Table 2, the comparison reveals similarity between the families COG3577 and Osvaldo, and between many Ty3/Gypsy families and SASPases and DDI eSNCAPs. The similarity between COG3577 and Osvaldo is significant but not sufficient to conclude recent horizontal transference in any direction, and the wide distribution of DDI eSNCAPs in plants, fungi and animals, suggests that, regardless of the relationship between this enzyme and *Ty3/Gypsy *LTR retroelements, this relationship is ancient. HMM analyses thus suggest a common ancestor for dimeric CAPs that, as a sequence, might have been an intermediate between SNCAPs and Ty3/Gypsy LTRCAPs. Here, an interesting question to address in further analyses is if the DTG/ILG template can be considered as a general consensus approximating the ancestral dimeric form.

**Table 2:** Comparison between HMMs and MRC sequences

COG3577 MRC sequence			DDI MRC sequence			SASPase MRC sequence		
HMM profile	Score	E value	HMM profile	Score	E value	HMM profile	Score	E value

Osvaldo	16.7	2.3e-05	NIX1	30.3	3.5e-08	Osvaldo	14.7	3.7e-05
COG5550	-5.1	0.019	412/Mdg1	16.0	5.7e-05	Reina	-2.3	3.2e-04
Caulimoviridae	-11.9	0.04	Athila	8.0	1.4e-04	Chrofung	-4.8	9.4e-04
Alpharetroviridae	-19.6	0.048	Del	7.1	2.6e-04	TF	-3.3	0.0025
Del	-17.5	0.051	Micropia/Mdg3	-2.7	8.5e-04	Del	-3.6	0.0025
Galadriel	-26.3	0.21	COG3577	-11.8	9.0e-04	Mag	-3.9	0.0093
Mag	-17.1	0.22	SASPase	-1.3	0.0024	Micropia/Mdg3	-14.0	0.012
DDI	-67.8	0.71	Osvaldo	-4.4	0.0034	Errantiviridae	-1.3	0.015
Lentiviridae	-25.4	0.87	Reina	-13.2	0.004	412/Mdg1	-9.0	0.043
			Chrofung	-11.5	0.0051	DDI	-57.8	0.076

The comparison was performed using the MRCs as queries to the HMM search at GyDB with hmmpfam. We only summarize the most significant (top) hits of similarity.

**Figure 4:**
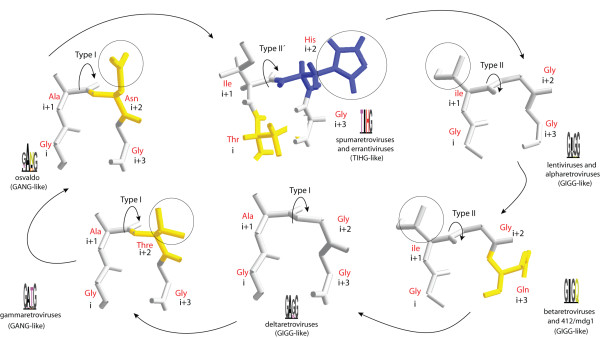
**Types of β-turns**. Based on 3 empirically characterized GIGG, GAGG, and TIHG flap-like β-hairpin loops, and 3 prediction GIGG and GANG models, the β-turns differ from each other based on the orientation the "Phi" and "Psi" torsional angles of the peptide bond between residues in "i+1" and "i+2" (indicated with curved arrows). Additional differences based on the properties of the residues in the position "i+1" are also observed (in circles). Chains were colored according to the physiochemical properties of amino acids. Hydrophobic residues are colored grey, acidic residues and relatives are yellow, and basic residues are in blue.

Taking into primary consideration that the different families preserve different pattern 3 motifs we have noted that the network can be interpreted evolutionarily and taxonomically. For instance, the *Retroviridae *is a group of retroviruses which can be divided in 3 taxonomical classes I, II, and II and we have also noted that there is a particular flap variant usually preserved depending on the class [18]. Since this point, another interesting aspect of the pattern 3 variability meriting further attention is to discern should each pattern 3 variant might be enclosing structural differences related to function. We explored this possibility combining empirical data with prediction models. That is, the flap is a β-hairpin loop consisting in 2 antiparallel β-strands coupled by a β-turn. The β-turn (also known as reverse turn) is a supersecondary structure originally described by Venkatachalam [43] that consists of 4 residues designed as "i", "i+1", "i+2", "i+3". In Figure 4 we illustrate 3 empirical examples of GIGG and TIHG β-turns based on the 4 amino acids that define the true flaps of the LTRCAPs encoded by HIV-1 [40], HTLV-1 [41,42], and SFV [39] retroviruses. Here, 3 types of β-turns appear to be distinguished according to prior classifications [44-46] based on the "Phi" and "Psi" torsional angles of residues at positions "i+1" and "i+2" (using the Ramachandran plot, data not shown). The TIHG turn apparently adopts the type II' conformation, while GIGG turns adopt the type II with the exception of the GAGG turn of deltaretroviral LTRCAPs, which adopts the type I. We think that this might be due to the alanine at "i+1" which has smaller side chain than that of the isoleucine "i+1" in GIGG turn. Figure 4 also shows 3 additional predictions based on the pattern 3 variations of osvaldo and gammaretroviral LTRCAPs (GANG-like), and betaretroviral and 412/Mdg1 LTRCAPs (GIGG-like) we modeled using the HIV-1 and HLTV-1 turns as templates. These 3 predictions are apparently consistent with the notion suggesting that GANG-like β-turns might adopt the type I conformation while GIGG-like turns implement the type II. Additionally, we performed β-turn type predictions using the different pattern 3 motifs summarized in Figure 3 as queries to the COUDES server [47]. For simplicity's sake we do not show the results obtained in this analysis but they were also consistent with the possibility addressed. Prediction models should be carefully interpreted because even empirically resolved structures may reflect constraints imposed by crystal formation rather than significant differences between the breathing native structures in solution. We can only speculate but the structural characterization of the different models merits further attention because predictions are sufficient consistent to discreetly arguing the possibility of structural differences based on pattern 3 motifs.

## Conclusion

The pre-existing classification combined with the evolutionary history of LTR retroelements permits a consistent taxonomical collection of sequence logos and HMMs. This set is useful for gene annotation but also a reference to evaluate the diversity of, and the relationships among, the different families. Comparisons among HMMs suggest a common ancestor for all dimeric CAPs that is halfway between SNCAPs and those coded by *Ty3/Gypsy *LTR retroelements. Sequence logos reveal how all clan AA families follow similar protein domain architecture related to the peptidase fold. In particular, each family nucleates a particular consensus motif in the sequence position related to the flap. The different motifs constitute a network where an alanine-asparagine-like variable motif predominates, instead of the canonical motif of the HIV-1 LTRCAP and closer relatives.

## Methods

### Sequences and database

We created a sequence database with 323 non-redundant CAPs collected from NCBI [48] and MEROPS [1]. Here, we would like to stress that concerning LTR retroelements, we used only sequences encoded by canonical sequences, almost all being functional. A more detailed summary of the sequences used is available in the Additional file 1, which provides an extended version of the guide tree illustrated in Figure 1. This tree summarizes names, taxonomy, hosts and Genbank accessions of all CAPs used.

### Alignments

We divided the 323 CAPs investigated in this study into 38 protein families according to prior estimations of taxonomy and evolutionary relationships. Then, we created 34 multiple alignments, one for each family having more than 2 sequences plus a single non-redundant alignment collecting all CAPs. Families were aligned using CLUSTAL X [49] and manually refined using the GENEDOC editor [50] in shaded mode. The refinement was conducted using the following groups of amino acid similarity: [T,S – small nucleophile amino acids], [K,R,H – basic amino acids], [D,E,N,Q – acidic amino acids and relative amides], and [L,I,V,M,A,G,P,F,Y,W – hydrophobic amino acids]. The non-redundant alignment was performed manually with GENEDOC and using the aforementioned groups of amino acid similarity. Here, we used prior structure-based alignments [4,20] as well as the information content shape delineated by the Andreeva's model [21,23] to establish a sequence core from which we excluded Andreeva's element A1 (because of the dissimilarity in this trait). We used this core to align and refine the different families manually, family-to-family. The refinement was led by inferring and using the clan AA phylogeny as a guide tree (the tree was inferred as many times we refined the alignment). This process revealed 6 amino acid patterns we call the DTG/ILG template because these 2 motifs predominate. This template is common to all CAPs investigated and was used as an anchoring criterion of alignment and refinement thorough the rest of this study.

### Ancestral maximum likelihood reconstructions

The different sets of AMLR sequences generated in this study were reconstructed using each family alignment as an input to FastML 2.02 [51]. This tool performs the analysis using 2 methods, Joint and Marginal [51-54] and reports 6 outputs – Pin, Fin, Jrof, Jpf, Mrof, and Mpf. The Pin output is an NJ tree [33] summarizing the relationships between CAPs (the input taxa) and AMLR sequences (the nodes). Fin is a file giving information about the parental relationship between AMLR sequences and CAPs. Jrof is the AMLR alignment obtained using the Joint method. Jpf is a file providing information about the joint probability per position and the total log likelihood in the Jrof alignment. Mrof is the AMLR alignment obtained using the marginal method. Mpf is a file giving the marginal probability per position and the total log likelihood in the Mrof alignment. All AMLR files are available online in CAARD introduced under "Results". There is a minor limitation when performing the AMLR analysis with FastML; should a particular CAP display an amino acid trait not found in the remaining CAPs, the tool includes such a trait in all AMLR sequences. To avoid this limitation, we processed the alignments containing these biases to remove the non-informative positions. The criterion was to eliminate alignment positions with 70–100% gaps and/or high amino acid entropy.

### HMMs

HMM profiles [55] were modeled and calibrated using HMMER [56] and the processed Jrof AMLR alignments as inputs. MRC sequences were derived from the HMMs using HMMER. All HMM profiles and MRCs are available online within CAARD introduced under "Results". The profiles can be consulted directly via the HMM search at GyDB, with the exception of the 4 HMM profiles describing the 4 pepsin domain, which are only available in the CAARD. In the HMM server, pepsins are represented by 2 HMM profiles, which describe the natural 2-domain forms of pepsins (sub-families A1A and A1B according to MEROPS [1]).

### Sequence logos

The sequence logo methodology consist in the creation of graphical representations of the consensus of DNA and protein multiple alignments. The methodology is useful to decipher the order of predominance and the relative frequencies of residues at every position. This provides a significant view of the patterns characteristic of a gene or a protein. In each position, each residue is a letter whose height is proportional to its frequency per position multiplied by the information content of each position measured in bits [57]. Letters are placed such that the most frequent is positioned at the top. In this study, sequence logo analyses involved CheckAlign 1.0 [58] in Shannon's algorithm mode [27] and correction factor. We used each processed Jrof AMLR alignment as an input to this tool. In each sequence logo, basic residues are represented in red, hydrophobic residues in black, amino acids that are common in β-turns (G and P) in dark grey, small nucleophiles in violet, acidic residues in orange and acidic-relative amides in green. CheckAlign directly builds the logo from an ungapped alignment using the conventional methodology [57,59]. Here, the maximum uncertainty by position in a protein alignment is log_2 _20 = 4.3. In the case of gapped alignments, CheckAlign builds the logo, taking the gap character as another amino acid species. Here, the tool considers the maximum uncertainty by position to be log_2 _21 = 4.4 for protein alignments. The different sequence logos constructed are available online within the CAARD introduced under "Results". CheckAlign also includes a naive relative-frequency algorithm we used to identify a preliminary form of the DTG/ILG template (for more details about CheckAlign see [58]).

### Comparative analyses

BLAST similarities were correlated using different queries (CAPs and MRCs) to the CORES database via the NCBI BLAST search [60] at GyDB using the BLASTp search mode. HMMs were tested using different HMM profiles as queries to the CORES database via the HMM search at GyDB using hmmsearch. We used the hmmpfam search tool in this server to compare the different MRCs with the HMM profiles, using the MRCs as queries to the HMM profile database available by default.

### Programming

The CAARD is a database presented through a web-based template programmed in PHP language [61]. The template retrieves information from the database through a management system based on URL parameters, and reproduces the radial topology of a phylogenetic tree inferred based on the alignment of all the processed Jrof AMLR sequences reconstructed. To perform this alignment we followed the same strategy used to obtain the non-redundant alignment using CAPs. The template acts as a presentation layer of the different datasheets stored, and cluster names depicted in the tree are links that invoke the stored information.

### Structural modeling

We illustrated the 3D structure of the HIV-1 LTRCAP using SWISS-PDB Viewer 3.7 [62] and the PDB file 1a30 [40]. The PDB file 1a30 was also used along with the PDB files 2b7f [41,42] and 2ijs [39] to illustrate with SWISS-PDB Viewer 3.7 the different types of β-turn types displayed in the flap of HIV-1, HTLV-1 and SFV LTRCAPs, respectively. All PDB files were downloaded from the RCSB Protein Data Bank [63]. We also used SWISS-PDB Viewer 3.7 for modeling the β-turn involving the GIGQ flap motif of betaretroviral and 412/Mdg1 LTRCAPs using the HIV-1 β-turn as a template. The GATG and GANG flap motifs typically found in gammaretroviral and Osvaldo LTRCAPs were modeled using the HTLV-1 β-turn as a template. The 2D structure prediction of the DTG/ILG template shown in Figure 1 was made using the PSIPRED server [64].

### Phylogenetic trees

The 2 phylogenetic reconstructions performed in this study – guide and database trees – employed PHYLIP 3.6 [65]. First, we generated 100 bootstrap replicates of each alignment using SEQBOOT. Second, we used the protein sequence parsimony method of Felsenstein, based on the approaches of Eck and Dayhoff [31] and Fitch [32], to perform the analysis. The bootstrap file was used as an input to PROTPARS and the input was randomized using the following parameters: random number seed = 5 and number of times to jumble = 5. Then, CONSENSE was used to obtain a MRC tree [66] using the tree file generated by PROTPARS as an input. As the MRC tree usually consists of all clusters that occur > 50% of the time, we took consensus values > 55 as a bootstrap reference. We used the bootstrap values to scale the trees. We also tested the NJ method [33] using different models of distances implemented in PROTDIST. Here, it is important to keep in mind that the overall efficiency of the different methods of phylogenetic reconstruction in building the true tree vary with substitution rate, transition-transversion ratio, and sequence divergence [67,68]. With the particular material we studied, phylogenies based on the parsimony principle proved themselves to be more consistent with the MEROPS classification and known LTR retroelement phylogenies than NJ trees. Parsimony trees also reported better bootstrapping and were more consistent with the comparative analyses than NJ trees. Nevertheless, NJ and parsimony trees were consistent to each other when the analysis was inferred using AMLR sequences instead of CAPs.

## List of abbreviations

(AMLR): Ancestral maximum likelihood reconstruction; (CAARD): Clan AA Reference Database; (CAP): Clan AA aspartic peptidases; (CIBERESP): CIBER de Epidemiología y Salud Pública; (DDI): DNA-damage inducible; (ENISA): Empresa Nacional de Innovacion S.A; (eSNCAP): Eukaryotic single-domain nonviral clan AA peptidase; (GyDB): Gypsy Database; (HMM): Hidden markov model; (HIV-1): Human Immunodeficiency Virus type 1; (HTLV-1): Human T-Cell Leukemia Virus type 1; (ICTV): International Committee on Taxonomy of Viruses; (LTR retroelements): retrotransposons and retroviruses with long terminal repeats; (LTRCAPs): LTR retroelement clan AA peptidases; (MRC): Majority-rule consensus; (ML): Maximum likelihood; (MuERV-L): Murine endogenous retrovirus-Leucine; (NCBI): National Center of Biotechnology Information; (NJ): Neighbor joining; (NIX-1): Neuronal interacting factor × 1; (ORF): Open reading frame; (pSNCAP): Prokaryotic single-domain nonviral clan AA peptidase; (PSSMs): Position-specific scoring matrices; (SFVs): Simian foamy viruses; (SNCAPs): Single-domain nonviral clan AA peptidases; (*Retroviridae*): Vertebrate retroviruses.

## Competing interests

The authors declare that they have no competing interests.

## Authors' contributions

CL and AM conceived and designed the study; CL performed the analyses; RF and GR programmed the database, and CL wrote the paper.

## Reviewers' comments

### Reviewer's report 1

**Reviewer 1: **Daniel H. Haft, J. Craig Venter Institute.

#### Reviewer's comment

This paper can be viewed as an archival reference to describe a web resource on clan AA aspartyl proteases. That family is of special interest because both HIV and eukaryotic LTR retroelements encode members of the family. The HIV protease is an important therapeutic target. The paper itself describes a work flow, but the pipeline does not correspond to a distributable tool because it includes essential manual steps. Instead, the resulting collection of sequence logos, HMMs, and web resources is the featured contribution of this work. There seem to be two main points of emphasis in this paper. The first point is that a pre-existing (but not yet complete) classification scheme for clan AA aspartyl proteases can be made into a self-consistent classification scheme by multiple sequence alignments, sequence logos, and HMMs. The classification includes both those subfamilies already explicitly defined in MEROPS and other implicit members recognized as belonging to the clan but awaiting explicit MEROPS treatment (e.g. COG3577 members). The second main point is that using Pattern 3 by itself, the "flap", leads to an informative classification scheme that distinguishes proteins according to motif-based matches in this glycine-rich beta-turn. The possibilities were not really discussed that convergent evolution, human bias during manual sequence alignment, or other alignment or interpretation troubles led the authors to produce a catalog of pattern 3 variants with relatively little value for guiding the interpretation of clan AA protease evolution. The pattern 3 motif catalog may turn out to have value for extrapolating protein function from characterized to uncharacterized members of the family. However, the reward to the reader from the pages-long description of how these "flap polymorphisms" support models for the evolution of clan AA may not justify the burden.

#### Authors' response

Yes, the flowchart combines software analysis and manual means to construct a set of tools including alignments, ancestral ML reconstruction analyses, sequence logos and HMMs. The classification is an improvement over the pre-existing classification but it is work in progress because of the large diversity of the eukaryotic LTR retroelements and their coded peptidases. For this reason, we created the database in order to establish a reference set that we plan to update in light of new data. Regarding the second point we have amended the manuscript discussing the possibilities addressed by the referee but have also toned down the most speculative aspects.

#### Reviewer's comment

The first draft of the paper is too long, with too much difficult syntax, paragraphs that are so long that the main points are hard to find, with too much speculative language. Review reports for the typical anonymous reviewer would likely require a substantial streamlining of the manuscript with improved clarity, necessitating a second round of review. I believe I must make an equivalent recommendation here. I find portions of the analysis to be novel enough and useful enough for eventual publication, and I find the web presentation of the same work quite nice.

#### Authors' response

Done, we have reduced the manuscript and figures. We have also revised the style and syntax, etc.

#### Reviewer's comment

I would like to note that unique identifiers (D.O.I. numbers) can be attached to electronic publications. Some content from original manuscript could be pointed to by your revised manuscript as a distinct document, and presented through the web site as free text or as database content, rather than being run through the peer review process as part of the work that reviewers are asked to endorse directly.

#### Authors' response

Done, we have separated the flowchart's description from the manuscript. This text and accompanying figures is now available as a section of the database.

#### Reviewer's comment

Note on justifying why the study of protein family evolution should be done: My perception is that, in a well-understood protein family with many 3-D structures, the insights to be gained by an evolutionary reconstruction are not nearly as great as when structural and mechanistic information is limited. By contrast, constructing a classification scheme that imposes a sensible nomenclature, and attaching the nomenclature to clear and useful functional description, does have value. A core of the paper here can fit that goal.

#### Authors' response

Done, we have rewritten the current manuscript version, which focuses on database/collection justifying the study in the basis of different arguments addressed in the manuscript introduction.

*Reviewer's follow-up comment based on second version*

#### Reviewer's comments

Following the authors' revision of the manuscript and response to comments, I have amended my comments somewhat. Some technical comments were fully addressed, are now irrelevant, and have been removed. However, I left some comments in place, even if largely addressed, as they represent a discussion of the work. This paper describes a body of work in which manual alignment editing, guided by extensive previous work in the field (crystallographic and interpretive), leads to consistent identification of six key motifs that unify a broad family of peptidases and clarify which of its features are general. Attempts to leverage from these human-curated subfamily alignments to develop additional useful database objects such as motif definitions, sequence logos, and phylogenetic trees, all presented through a smoothly functioning web site, have met with some success, and the work is expected to continue past the date of publication for this work. Portions of the discussion in the article may be regarded as somewhat speculative.

#### Authors' response

We thank this expert in the field of protein families for his constructive criticisms and positive assessment of the issue and database site.

#### Reviewer's comments

The discussion of flap motifs seems somewhat cleaner and clearer, but it still mixes in considerable speculation and is still quite long. The sequence logo, a one-dimensional representation of consensus sequence, may be overworked here as a tool for providing insight into protein 3-D structural variation near the protease active site.

#### Authors' response

By comparing all sequence logos deposited in CAARD, one can easily visualize how all sequence logos exhibit similar information content shape. The information content shape of each sequence logo is defined by the number of computed bits for which Shannon's algorithm provides a information measure for each amino acid or symbol (since information is statistically random and each new symbol would increase the measurement). This means that a sequence logo provides information on not only the consensus sequence of a DNA and/or protein family but also other information related to natural patterns (see Schneider [26]). In this case, the different patterns and information content shape of the clan AA protein domain architecture (in general) appear to be delineated by the peptidase fold. In this study, we started using a structure-based model based on retropepsins (previously resolved and widely supported by a number of studies). What we have found is that the remaining clan AA families are consistent with this particular information content shape and that the structural model (i.e. the Andreeva's template) can be resolved as a sequence template which we divide into 6 amino acid patterns (the DTG/ILG template). Here, the equivalence between pattern 3 and flap is clearly supported by the 3D structure of all empirically characterized aspartic peptidases (or aspartyl proteases). Due to the high degree of conservation of pattern 3 in almost every, but not all, remaining CAP families evaluated, one can certainly assume that pattern 3 is functional and that the likeliest and most reasonable function for pattern 3 is flap (by position and by motif similarity to the true empirically characterized flaps). We do not argue that all members within many families are carriers of flaps since the degree of conservation of pattern 3 varies in many cases and because there is evidence of flap lost (in the case of the pepsin lobe 2). We have presented a collection of pattern 3 variants, which are preserved depending on the protein family and prior data indicates that they can be related with the flap. This finding should be addressed as a result and we think that the speculation is justified as part of discussion. However, we have re-written the final version taking particular care to refer to only as flap those pattern 3 motifs having empirically demonstrated equivalence. In turn, we refer to the collective of positional traits which are equivalent by position, degree of preservation, and motif similarity to the flap as pattern 3.

#### Reviewer's comments

While certain structural elements may be important for nucleating folding, I would expect proteins to have some elements for structure and some for specifity. Since the flap element can flip right off the structure, and is known to be involved in binding substrate, it must be other beta turns that nucleate folding. The whole text from "This arrangement is a preliminary display" to "had selective advantages from this feature" probably should be removed.

#### Authors' response

Amended, we have removed the most speculative comments (those related to nucleating folding) from the text addressed above, and we tone down the comments that we think are justified, well supported, or needed as a discussion.

#### Reviewer's comments

I rebel against the idea that, in a family of proteins so divergent no automated alignment is possible, "the GAGG motif is a transitional state between the GIGG and GANG variations." So many factors affect the definition of these motifs – manual alignments, founder effects from nucleating each motif, co-evolution with the rest of each protein – that even the level of speculation that remains seems excessive. The current figure 4 discusses 3D structure of the flap based in part on 3 actual structures and 3 predicted structures, without making it clear enough which is which. The value of this analysis is highly questionable.

#### Authors' response

We agree with the referee regarding the fact that the automated alignment of extremely divergent protein families is possible. However, we did not find any algorithm or tool capable of exhaustively aligning more than 2 or 3 non-redundant clan AA families (and in all cases the automated alignment needed manual refinement). Note that the difficulty of clan AA is not to obtain an exhaustive alignment but to exhaustively align all its possible sequence forms. So we think that the manually performed model in this study can be useful as a case study to investigate new automations for exhaustively aligning fast evolving proteins. Upon this, our model considers 6 amino acid patterns which appear to be preserved in terms of physiochemical properties of amino acids in every evaluated clan AA family. The 6 patterns provide a sequence core to which other sequences have shown significant similarity hits in the following performed comparisons: 1) between sequences within families, and 2) between family consensus sequences and/or HMMs. This includes the general DTG/ILG template HMMs in its 2 current forms (with or without Ty3/Gypsy sequences). The information about similarity is available online in CAARD as the Supplementary Table P1 accompanying the flowchart section available in the URL [19]. The different similarities displayed in this Table correspond to the different pairwise alignments available in the "Section Models" of CAARD between the DTG/ILG template model and each family consensus. By evaluating these alignments, one can see how all consensus sequences constructed based on all dimeric CAP families align pattern3-to-pattern3 with the DTG/ILG template. This is because pattern 3 is a positional trait, essential in order to anchor the core of similarity between 2 CAP sequences. On the other hand, we have noted that clan AA can be automatically aligned into subsets of 2, 3 or even 4 families. For instance, it is known that the *Retroviridae *is a group of vertebrate retroviruses whose encoded LTRCAPs can be automatically aligned using software tools. In all cases, the different GANG, GIGG, and TIHG motifs of the Retroviridae LTRCAPs usually align pattern3-to-pattern-3 without needing manual refinement. This is because the trait we have called pattern 3 is strongly preserved in the different LTRCAPs encoded by the different Retroviridae genera. One can find similar result when aligning many other CAP families or when aligning other families with Retroviridae LTRCAPs. This means that there is no human bias in the definition of patterns 3 in the families exhibiting high degree of preservation. Another question is, can this pattern be labeled as flap? At this point, regarding dimeric CAPs, the empirical prior information is consistent with this possibility but, following the words of caution addressed by this referee, we have toned down to avoid excessive speculation. The highly preserved patterns 3 present a wide range of motif variability that can be used to identify other patterns in the less preserved families. In these cases of less preserved families, we recognize that the information content enhancement of sequence patterns may be biasing the pattern 3 motif towards the most frequent motif in these families and many other sequences within these families may be carriers of other motifs or have lost the flap. We have discussed more widely this possibility in the manuscript. Finally, we agree with this referee that there are many aspects that probably affect the network of flap polymorphisms such as co-evolution with not only the rest of each protein but also other proteins (in the case of eukaryotic LTR retroelements). Nevertheless, whatever the cause of the different pattern motifs found in the network, we think that it is clear that there is an evolutionary constraint involving the variability of pattern 3 since each CAP family usually preserves a particular motif (the feature can be used for taxonomy purposes). From that point on, we have tried to additionally explore the structural meaning of the variability of pattern 3 by comparing true flap β-turns and using them to make a predictive analysis. Predictions are analyses usually used for publication purposes and we present Figure 4 in these terms. However, we have re-written the paper clarifying the limitations of this Figure.

### Reviewer's report 2

**Reviewer 2: **Vladimir Kapitonov, Genetic Information Research Institute, Mountain View

#### Reviewer's comment

The authors have developed a database of the AA clan of aspartic proteases. Although the AA clan was included previously into the MEROPS database, a general database of proteases, its enormous diversity clearly justifies development of a more detailed database similar to the one described in this paper. The authors have mentioned that Bel aspartic proteases are not described in MEROPS as a separate group of aspartic proteases. Actually, a few years ago, there was so called A17 family of Bel/Pao peptidases in MEROPS. Moreover, the MEROPS A17 peptidase family was also introduced into Pfam (PF05380), which is actively used at NCBI for annotation of protein domains. However the A17 family was described wrongly as the protease. In fact, this was a ribonuclease (RNase H). Later, the A17 family was excluded from families of proteases in MEROPS. Yet it is still reported as a protease in Pfam and GenBank protein domain annotations. So I am sure that the establishment of a separate properly curated database of the AA proteases will help to avoid such problems.

#### Authors' response

We thank this expert in the field of mobile genetic elements, for his positive feedback and comments for improving the original and future approach. Certainly, sequencing projects have revealed how the diversity of clan AA greatly exceeds the current classification. We agree that the large diversity of eukaryotic LTR retroelements and the need to clarify their relationships with their host counterparts justifies the creation and continuous update of a curated database.

#### Reviewer's comment

My main concern is a disproportionally high number of Gypsy families introduced in the database. Among 38 families of the AA proteases, 17 families belong to Gypsy LTR retrotransposons! I understand that the authors have started this project working on Gypsy. However, other groups of LTR retrotransposons, including Copia and BEL, are as diverse as Gypsy: and yet each of them is represented here by a single family. As a result, general evolutionary reconstructions and scenarios made based on such biased data would be unreliable. The authors wrote (page 13, last paragraph) that the variability of BEL and Copia proteases is lower than that of their Gypsy and Retroviridae counterparts. Is that so? Just a brief look at Copia proteases via PSI-BLAST shows that these proteases are as diverse as Gypsy! After a few rounds of PSI-BLAST iterations, one can easily collect numerous Copia proteases less than 17% identical to each other.

#### Authors' response

Certainly, our study improves the pre-existing classification but the diversity of clan AA is extremely large to be completely addressed in a single study. In particular, the peptidases encoded by LTR retroelements are extremely difficult to deal with because of their diversity, conflicting signal, and possibility of being part of pseudogenized transposable copies. For this reason the database is in continuous progress. In this first version, we paid special attention to Ty3/Gypsy and Retroviridae peptidases because we previously exhaustively analyzed these 2 groups of LTR retroelements based on not only on the peptidase domain but also other protein domains such as gag, RT, RNAse H, INT, etc. What we found is that the phylogenetic signal of the peptidase domain of Ty3/Gypsy and Retroviridae elements is low but consistent with their differentiation into clades and genera. Along these lines, sequencing projects continuously reveal new lineages belonging to the different LTR retroelement groups. Therefore we think that the Ty3/Gypsy and Retroviridae families summarized are not disproportional; they are based on the clades and genera of LTR retroelements we currently classify. As the matter of fact, there are Ty3/Gypsy lineages such as those Gmr1 and Tor-like that are not included here and whose peptidases deserve analysis and classification. However, the referee is right when noting that other LTR retroelement groups such as *Ty1/Copia *and *Bel/Pao *are as diverse as *Ty3/Gypsy *LTR retroelements. Here, we are committed to perform identical investigation based on these (and other) retroelement groups. However, because of the large material to investigate, this is a separate study on which we are currently working because while there are previous studies focusing on the *Bel/Pao *and *Ty1/Copia *groups there is not yet a comprehensive phylogenetic differentiation of these groups into clades and genera. It is true that there is an existing agreement of classification to divide Ty1/Copia elements into several *Pseudoviridae *genera, but these genera are based on priming mechanisms instead of the phylogeny. Despite this, the current differentiation into families achieved in this study is not particular bias against *Ty1/Copia*, and *Bel/Pao *peptidases (in terms of phylogenetic signals). Unlike Ty3/Gypsy peptidases, the different Ty1/Copia and Bel/Pao peptidase sequences usually fall in independent single clusters in phylogenetic analyses because they have particular features not found in other peptidases (we have included some discussion in the manuscript). For this reason we have followed the MEROPS approach to classify these 2 into 2 families. Despite this, we are in complete agreement with this referee with regard to the fact that Ty1/Copia and Bel/Pao sequences must be taxonomically classified into a more complex classification into families, a topic we will focus on in further updates and that will probably reveal very exciting insights.

#### Reviewer's comment

Given the enormous sequence data available, the authors don't have to limit themselves by only experimentally studied retrotransposons. They can use protease sequences encoded by consensus sequences of hundreds of young families of LTR retrotransposons collected in other databases (e.g. Repbase). It would be beneficiary to the community if each family of the AA proteases in this database will be accompanied by a separate PSSM available to users. Very often, PSI-BLAST is as good as HMM. Therefore, the available PSSM would be helpful in PSI-BLAST-based detections of AA proteases.

#### Authors' response

In principle and in order to create phylogeny-based HMMs, the Gypsy Database (GyDB) project follows the phylogenetic analysis and database annotation of non-redundant canonical sequences with the full genome completely available. That is the scope of the GyDB, not to exhaustively align all sequences but look for reference models in a context of non-redundancy. About this, we find the 2 ideas proposed by this referee very interesting and we are certainly open to collaborate with the REPBASE team and/or use material retrieved from this senior database to implement not only the peptidase database but also other sections of the GyDB project. Moreover, we are currently working to implement the GyDB with wiki software to let other authors and research groups to contribute material of any kind, server and tools. Upon this scenario, the possibility to implement the clan AA database with PSSMs is viable and compatible with our classification criterion if using the MRCs currently available to iterate searches that can improve the capability of the phylogenetic HMMs or generate highly informative PSSMs. We think that this is important because of the conflicting signals found between several families. Our reasons about this criterion are directly related with the following Reviewer's comment and our answer.

#### Reviewer's comment

I am quite skeptical about using currently available tools and methods (including Joint and Marginal, mentioned by the authors) for reconstruction of ancestral AA proteases. Most of these proteases were/are encoded by transposable elements. Different families of transposable elements, regardless of their proteases, can differ dramatically from each other in terms of their transposition rates. Families that were more successful in massive transposition are represented by higher numbers of copies in genomes than their slow-transposing relatives. Usually, one collects randomly a number of different families. Therefore, any standard reconstruction of an ancient protease ancestral to proteases encoded by these elements would be biased towards families that had high transposition rate. The more ancient ancestry would one try to reconstruct the more significant transposition related bias will be.

#### Authors' response

Following the indications of referee 1, we have derived the manuscript to its most immediate scope – the collection of sequences and its uses. Along these lines, our main intention when using ancestral ML reconstruction in this study was to enhance the information content of each family instead of reconstructing the ancestor (we emphasized in the manuscript that the probability of reconstructing the true ancestor is near zero). Certainly, not all clan AA families need this kind of process but when dealing with this enzyme group, one finds different degrees of preservation. In many cases, the information content of many families is certainly low probably because of the points addressed by this referee, and software fail to construct informative logos and HMMs. However, we have tested the collection in many ways, one of them being the screening of a genome project with HMMs where they proved excellent for annotating new sequences. All queries detected peptidase sequences that were later confirmed to belong to the LTR retroelement lineage related with the HMM used. So the collection works, this means that the difficulties posted by the referee are precisely the same reasons that motivated us as to make such an exhaustive analysis of the different monophyletic families of peptidases (taking into account their evolutionary relationships and prior taxonomy). This difficulty was also the reason why we used ancestral ML reconstruction to emphasize the most prominent domain architecture of each monophyletic dataset. The analysis is not affected by the different rates of evolution because what we characterized is the clan AA protein domain based on all possible phylogenetic signal and not the functional families. The main basis of this argument is that the HMM describing the peptidases encoded by a particular LTR retroelements lineage will be able to show the best similarity to their lineage counterparts, and so on. Note as an example the peptidases encoded by the 412/Mdg1 and Micropia/Mdg3 LTR retrotransposons, which are more similar to those of vertebrate retroviruses (retropepsins) than to those of other Ty3/Gypsy elements. At the same time these peptidases are not similar enough to those of vertebrate retroviruses to conclude recent recombination and/or horizontal transference, and despite this, they show similarity to other Ty3/Gypsy peptidases. Obviously, the diversity of several peptidases may escape detection of the current HMMs but the classification is in progress and new sequences will calibrate the set of tools. On the other hand, the set of tools and combination of methods gives various angles for investigating different aspects of clan AA.

#### Reviewer's comment

One minor comment is on terminology. The authors use regularly terms "cohort", "families", "pools" making the manuscript unreasonably convoluted. "Cohorts" can be easily replaced by "families"!

#### Authors' response

Done, we have replaced the term "cohort" by "family".

#### Reviewer's report 3

**Reviewer 3: **Ben M. Dunn, University of Florida College of Medicine, Department of Biochemistry and Molecular Biology

#### Reviewer's comment

As for my comments, I offer the following from the perspective of an enzymologist who has worked on both the retroviral members as well as the eukaryotic members of the AA family. The author's effort to derive relationships between the various members of the AA family of peptidases is important and overdue. Natalia Andreeva constructed a "structural template" for the pepsin-like enzymes back in 1990 and presented this at a conference in Sonoma, California (published as reference #6 of the manuscript). She related the sequence of HIV-1 protease to the pepsin family and showed that retroviral sequences could also fit into the structural template. Ten years later, Wlodawer and Gustchina (who was Andreeva's graduate student) presented a thorough analysis of retroviral protease properties, including demonstration that additional structural data confirmed and expanded Andreeva's model of the "fold" of all enzymes in the AA family. However, the absolute identity of amino acids between the 2 groups of peptidases is very low, making it very difficult to make conclusions about similarities. Thus, the work of Llorens and colleagues is important in pushing the analysis as far as possible.

#### Authors' response

We thank this expert enzymologist for his positive review. Indeed, clan AA is one of the most widely studied enzymes in the scientific literature but it is at the same time one of which little is known because of its large diversity. While enzymes such as pepsins and retropepsins have been extensively investigated, others remain yet uncharacterized. The comment of this referee is indispensable to evaluate the object of the bioinformatic flowchart. We constructed an ongoing database to investigate and classify the different clan AA families using a major consensus derived from Andreeva's model to understand the different patterns (imprinted over sequence by the peptidase fold). With this aim, we continued in the same direction as prior approaches. Here, we investigate what is common between not only extensively studied aspartic peptidases such as pepsins and retropepsins but also others of which little is known.

#### Reviewer's comment

From a bioinformatics perspective I think that this is a good and carefully designed work that tries to impress by an amount of analyses whose significance and effectiveness is very hard to evaluate. Figures and the interactive website look very nice, professional, and probably useful to researchers in the field. The authors do a very meticulous job in aligning the sequences, finding conservation patterns, searching databases and creating evolutionary trees with different methods and outputs. In my opinion the bioinformatics of what they do is not particularly innovative or creative and I think they do a lot of over-killing but at least they are very exhaustive in their analysis. One aspect of the paper that disturbs me a little is that they seem to make a huge effort to demonstrate exceptional depth and insightfulness in their analyses. I am not sure what they are actually gaining by going through those complex exercises of finding hmm profiles, anchoring alignments, etc etc. I was never sure of what was accomplished at each step of the analysis and I had often the impression that a lot of dust was being raised. This impression may also be a reflection of the fact that the authors use a pompous and often obscure language. I would ask to lighten up the language a lot before publication

#### Authors' response

Certainly this issue is difficult because it involves 3 different areas of research – enzymology, LTR retroelement evolution, and protein families. We have carefully rewritten the manuscript to make the language accessible to any reader. We hope that this expert will now find the improved manuscript straightforward. Briefly, the key to understand why so many analyses, has been addressed by this referee with the sentence.

"The absolute identity of amino acids between the 2 groups of peptidases is very low, making it very difficult to make conclusions about similarities."

If this is the case with just 2 families, one can only imagine what happens when evaluating 3, 4 and more families. Our objective was not to impress but to demonstrate the significance of our results. There are various problems when dealing with clan AA. In fact, this enzyme group is a conundrum that cannot be resolved with elegant analyses but by doing a lot of work usually in the border of the significance, which should be therefore confirmed by various analyses. Upon that, the set developed in this study works, we tested the different HMMs and MRCs with a genome project, where they proved excellent to detect and annotate new sequences. However, as the point we see the large diversity of clan AA, each protein family is a particular case to study and the study of all cases gives the background to evaluate the whole enzyme group. Our database project describes different tools (and will probably describe more, see our response to referee 2) which can be used in many aspects. However, it is also a long term research where we investigate the origins and diversity of clan AA. To have significant results we should made different analyses in different ways. For instance, the only way to resolve by significant means the DTG/ILG template, as a logo as an informative profile, was by anchoring the MRCs and reconstructing various intermediate states by AMLR. The whole pipeline is a daunting task, but it has given as various clues to programme several scripts to process and align not only clan AA but also other fast evolving protein families. We have rewritten the manuscript with a more appropriate language and have removed unnecessary material as much as possible such as the former Tables of BLAST statistic included in the database (to show that the different HMMs and MRCs were tested). We hope that this additional correction will give a more appropriate and easy-to-use aspect to the database. See also our response to referee 2.

#### Reviewer's comment

P. 10: I don't understand what the following means: "The analysis revealed a process of evolutionary divergence organized hierarchically into a natural network from prokaryotes to eukaryotes".

#### Authors' response

The analysis refers to the qualitative comparison of the sequence logos, which at the same time are computational results by themselves. Logo methodology assigns size to each amino acid species in the graphical representation. This is by using Shannon's algorithm to estimate the entropy of each amino acid species per alignment position, and according to information theory. The most important trouble to follow this issue is that it compresses so many topics into a single manuscript. The referred network is obvious for an expert working in LTR retroelement evolution because knows the bio-distribution and differentiation of the LTR retroelement groups into lineages (clades, genera, etc). In fact, the network was previously reviewed and introduced regarding Ty3/Gypsy and Retroviridae LTR retroelements in a prior study [18]. In this clan AA manuscript, we show that the network also extends to other LTR retroelement groups and other peptidases. The different motifs are similar but not identical. The hierarchy from prokaryotes to eukaryotes comes from the fact that motifs can be established in variants establishing different relationships among the different families.

### Reviewer's follow-up comment based on second version

#### Reviewer's comment

The figures are improved, although I still cannot read all the text in Figure 2. I know that this from a screen shot, so I guess it is okay. The one remaining problem is that your manuscript is very much of a "mid-term" progress report. It suffers from the fact that it does not describe a finished product. This is probably okay in your field, so I don't want to make a big point about this.

#### Authors' response

The analysis of clan AA is work in continuous progress. For this reason the main focus of the manuscript is the database tool and the flowchart/pipeline. This manuscript will be the citation reference for further updates of the database even if we publish additional data (in further updates we will not describe the tool just the results in further approaches). This means that the research is in-progress but the database (as version 1) is job completed.

## Supplementary Material

Additional file 1 **Clan AA phylogeny used as guide tree**. By clicking the name of each OTU in this tree, the user can locate a file at GyDB providing information about the sequence selected, including a link to its Genbank accession at NCBI. If the sequence has no GyDB file, the links takes the user directly to the Genbank accession. Click here for file

Additional file 2 **Clan AA aspartic peptidases: alignment URLs**. All alignments performed in this study are freely available online. This table summarizes and provides links to the different alignments URLs. By clicking the name of each alignment, the user can locate the alignment in various formats. By default, the alignment is presented in a shaded format that facilitates visualization of the sequence patterns, and provides links to the Genbank accession of the different sequences aligned. Click here for file
