# Supplementary material for: Bioinformatic flowchart and database to investigate the origins and diversity of Clan AA peptidases
Source: Biol Direct. 2009 Jan 27;4:3. doi: 10.1186/1745-6150-4-3 (PMC2642776; doi:10.1186/1745-6150-4-3)
Supplement: Additional file 2 — Clan AA aspartic peptidases: alignment URLs. All alignments performed in this study are freely available online. This table summarizes and provides links to the different alignments URLs. By clicking the name of each alignment, the user can locate the alignment in various formats. By default, the alignment is presented in a shaded format that facilitates visualization of the sequence patterns, and provides links to the Genbank accession of the different sequences aligned. [file 1745-6150-4-3-S2.zip › Additional_file_2.htm]

Additional File 2


|  |  |  |
| --- | --- | --- |
| **Additional File 2. Clan AA aspartic peptidases: alignment URLs** | | |
| Taxonomy | Alignment links | Sequences |
| Retroviridae LTRCAPs | Lentiviridae | 11 |
|  | Alpharetroviridae | 3 |
|  | Betaretroviridae | 8 |
|  | Gammaretroviridae | 13 |
|  | Deltaretroviridae | 4 |
|  | Spumaretroviridae | 6 |
| Ty3/Gypsy LTRCAPs | 412/Mdg1 | 2 |
|  | Athila | 9 |
|  | Cer2-3 | 2 |
|  | Chrofung | 14 |
|  | CsRN1 | 2 |
|  | CRM | 3 |
|  | Del | 7 |
|  | Errantiviridae | 14 |
|  | Galadriel | 3 |
|  | Mag | 7 |
|  | Micropia/Mdg3 | 3 |
|  | Osvaldo | 4 |
|  | Reina | 4 |
|  | Tat | 10 |
|  | TF1-2 | 2 |
|  | Ty3 | 3 |
| Other  LTRCAPs | Bel | 16 |
|  | Caulimoviridae | 18 |
|  | Ty1/Copia | 26 |
| pSNCAPs | COG5550 | 10 |
|  | COG3577 | 20 |
| eSNCAPs | DDI | 20 |
|  | NIX-1 | 5 |
|  | SAPases | 6 |
| Pepsins | 2-domain form | 27 |
|  | 2-domain form | 5 |
| clan AA | Non-redundant alignment | 323 |
| MRCs and others | DTG/ILG template | 38 |
